# An Attempt to Establish the Consensus Regarding the Diagnosis, Classification, and Treatment of Rosacea in Japan Using a Modified Delphi Method: The Japan Rosacea Consensus

**DOI:** 10.1111/1346-8138.70213

**Published:** 2026-03-12

**Authors:** Kenshi Yamasaki, Nobukazu Hayashi, Hiroaki Hayashi, Yasuko Fukuya, Yuki Horiuchi, Miwa Kobayashi, Yoshimasa Nobeyama, Jun Omatsu, Kanako Tsunoda, Yoshiki Miyachi

**Affiliations:** ^1^ ALOOP Clinic & Lab Tokyo Japan; ^2^ Department of Dermatology Toranomon Hospital Tokyo Japan; ^3^ Hayashi Skin Clinic Kobe Japan; ^4^ Department of Dermatology Nerima Hikarigaoka Hospital Tokyo Japan; ^5^ Akihabara Skin Clinic Tokyo Japan; ^6^ Kobayashi Dermatology Clinic Kitakyushu Japan; ^7^ Department of Dermatology The Jikei University School of Medicine Tokyo Japan; ^8^ Department of Dermatology University of Tokyo Graduate School of Medicine Tokyo Japan; ^9^ Department of Dermatology Iwate Medical University School of Medicine Shiwa Japan; ^10^ Shizuoka Graduate University of Public Health Shizuoka Japan

**Keywords:** consensus, Delphi method, dermatologists, differential diagnosis, rosacea

## Abstract

In Japan, rosacea has attracted increasing interest. However, because rosacea had been thought to be relatively rare in Japan, the perception of this disease varies among dermatologists. To address these challenges, this study developed a consensus regarding the diagnosis, classification, and treatment of rosacea using a modified Delphi method based on the expertise of Japanese specialists in rosacea. Ten Japanese dermatologists were included in the expert panel based on their expertise in rosacea treatment and contributions to the field of rosacea. For each item that mentioned rosacea, the specialists responded with “disagree,” “neither agree nor disagree,” “agree,” or “don't know/can't answer.” Consensus was defined as ≥ 80% agreement among panel members. Panel consensus was obtained for all 50 items related to rosacea (e.g., disease types, characteristics, diagnosis, factors for onset and exacerbation, and treatment). An online survey on the consensus statements revealed discrepancies between general dermatologists and panel members. In the postsurvey meeting, the panel members discussed the differentiation between rosacea and similar diseases and proposed an algorithm for differentiating rosacea. The panel members concluded that the goal of rosacea treatment in Japan should be to “maintain a state wherein the patient's daily life is not affected by symptoms over the long term” rather than “complete cure.” Thus, this study integrated the findings of the expert panel and proposed a consensus statement on rosacea treatment in Japan. The organization of knowledge and dissemination of information regarding rosacea among general dermatologists will improve the treatment outcomes of patients with rosacea.

## 1 Introduction

Rosacea is a chronic inflammatory disease that often occurs in adults over the age of 30 years. It is characterized by flushing, persistent erythema, papules, pustules, and telangiectasias. These symptoms can be triggered and exacerbated by temperature changes, ultraviolet radiation, spicy food, alcohol, and physical exercise [1]. Moreover, they can significantly affect a patient's quality of life [1]. The onset of rosacea is associated with an overactive immune system [2] and abnormal regulation of the neurovascular system [3]. Both genetic and environmental factors contribute to the development of the disease [4]; however, its pathogenesis remains unclear. Rosacea cannot be diagnosed using physiological and molecular indicators. Currently, the diagnosis of rosacea is based on the visual examination of clinical symptoms, patient interviews, and detection of exacerbating factors. Consequently, patients with rosacea may be undiagnosed, leading to the underestimation of its prevalence in Japan.

The National Rosacea Society proposed a subtype‐based classification of rosacea in 2002, which included four subtypes: erythematotelangiectatic, papulopustular, phymatous, and ocular [5]. As this classification does not accurately capture all clinical characteristics of rosacea, a phenotype‐based classification that includes the main symptoms, individual features, and environmental factors is warranted. To reflect this shift in perspective and provide appropriate treatments for patients, the ROSacea COnsensus (ROSCO) panel was established [6]. The ROSCO panel developed a global consensus regarding the diagnosis and management of rosacea using the Delphi method. After the ROSCO 2017 consensus was published, the use of the phenotype approach to diagnose rosacea increased [7].

In Japan, the treatment guidelines were revised in 2023 to follow the international trend of rosacea classification [8]. However, several challenges related to the diagnosis and classification of rosacea remain unaddressed in clinical practice. For example, the percentage of rosacea or rosacea‐like dermatitis was estimated to be 0.22% among patients who visited a dermatologist in Japan [9] though a previous meta‐analysis estimated that the international prevalence of rosacea among dermatological outpatients is 2.39% [10]. Although a direct comparison cannot be made, this discrepancy can be attributed to the following reasons: (1) the lack of consensus on the diagnostic criteria and measurement in Japan, leading to inconsistent diagnoses [11], and (2) difficulty in detecting erythema and telangiectasia in the skin of people of color, including Japanese [12]. With regard to the latter, since patients are unaware of their symptoms, it may lead to reduced opportunities for patients to visit dermatologists and hinder accurate diagnosis [12, 13]. Another challenge in Japan involves the complexity of defining and differentiating steroid‐induced rosacea, rosacea‐like dermatitis, perioral dermatitis, and rosacea. This ambiguity of disease classification makes it difficult to diagnose rosacea in the clinical setting in Japan.

In this context, a consensus on the diagnosis, classification, and treatment of rosacea is needed to fully take into account the skin type of Japanese people and the level of dermatologist understanding of the circumstances relating to rosacea in Japan. Therefore, to develop consensus statements for rosacea in Japan, the present study gathered and integrated the expertise and experience of Japanese specialists in rosacea.

## 2 Methods

### 2.1 Study Design

**FIGURE 1:**
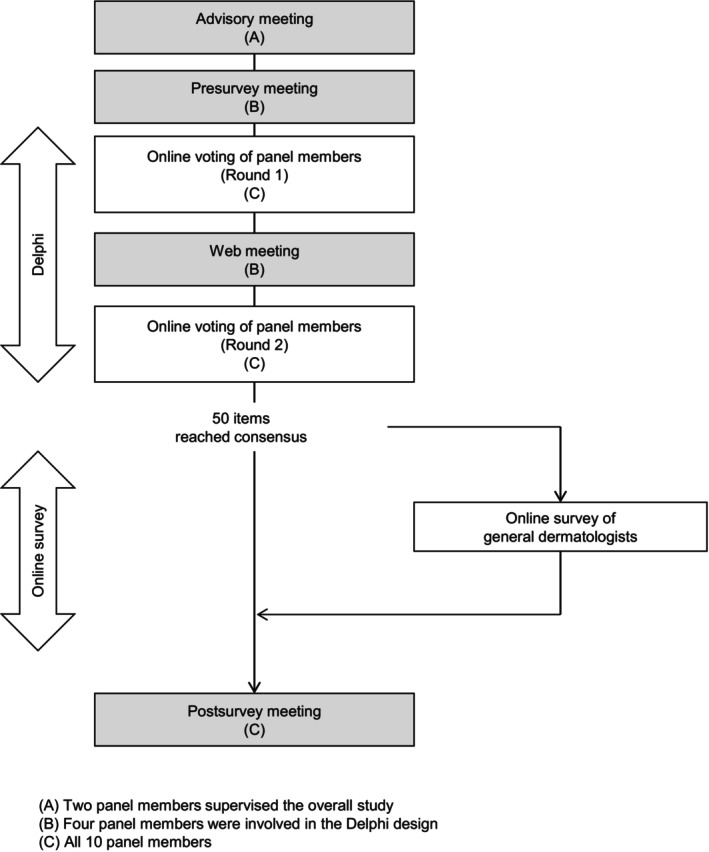
Study design. The overall flow of the study is shown. Gray squares indicate the meetings conducted by the panel members, whereas white squares indicate the online voting of panel members or the online survey of general dermatologists. The letters (A, B, and C) beside the squares indicate the groups of panel members involved in the process. The steps that correspond to the Delphi method include the steps from the presurvey meeting to the online voting of panel members (Round 2).

Consensus on rosacea was developed using a modified Delphi method. The Delphi method involves experts assessing complex issues in an iterative and structured process to develop a consensus [14]. Moreover, it is highly useful for building consensus in fields that lack evidence [15] and has been used to establish various clinical guidelines [14, 16]. The flow chart of this study is shown in Figure 1. The design of the Delphi method and expert panel members were decided at an advisory meeting. In this study, the modified Delphi method included two voting processes and two meetings, and some items with no consensus in Round 1 were revised through an online meeting. After two rounds of voting, 50 items were identified through consensus. Additionally, an online survey was performed to evaluate the current understanding and management of rosacea among general dermatologists in Japan, although this step is not typically included in the Delphi procedure. The online survey involving physicians who regularly treat rosacea (*n* = 216) assessed the level of agreement with 22 statements on the basic management of rosacea, which were selected from 50 consensus statements. The 10 panelists who participated in developing the consensus did not participate in the online survey. Finally, a postsurvey meeting involving the panel members was performed to discuss the results of the two rounds of consensus development and online survey.

### 2.2 Expert Panel

The expert panel who were selected during the advisory meeting comprised 10 Japanese dermatologists. The members of this panel were selected based on their contributions to the field of rosacea, their expertise in rosacea treatment, and the type and region of the facility to which they belong. These 10 dermatologists participated in two rounds of online voting and a postsurvey meeting. Four of the 10 panel members were involved in the design of the Delphi method and creating the draft items. Among them, two supervised the entire process. Their demographics are as follows. All expert panel members were dermatologists certified by the Japanese Dermatological Association, including four affiliated with clinics, four with university hospitals, and two with general hospitals. The locations of these facilities were scattered throughout the Tohoku, Kanto, Chubu, Kansai, and Kyushu areas.

### 2.3 Development and Administration of the Survey

The questionnaire items were constructed to assess the level of agreement using terms such as “disagree,” “neither agree nor disagree,” “agree,” and “don't know/can't answer.” Voting was anonymous. Consensus was defined as the agreement of ≥ 80% among panel members. Specifically, the agreement rate was calculated using the number of “agree” votes as the numerator and the total of 10 votes, including those categorized as “don't know/can't answer,” as the denominator. In addition, each item included an open‐ended question. The opinions expressed in the open‐ended questions were used to revise the items and build consensus. To maintain anonymous voting, Social Survey Research Information Co., Ltd., which was not involved in developing the consensus, managed the voting processes and performed the descriptive analysis of the results.

### 2.4 Extra Research: Online Survey for General Dermatologists Treating Patients With Rosacea

**FIGURE 2:**
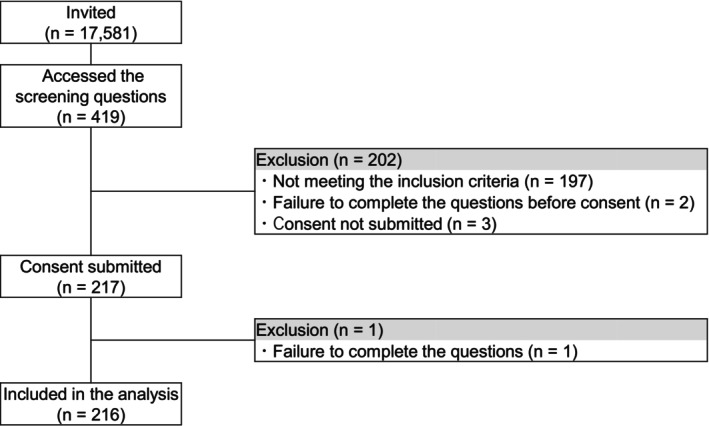
Flowchart showing the selection process from physicians for the online survey of general dermatologists.

The survey targeted physicians (excluding medical residents) in the dermatology or plastic surgery departments who had treated two or more patients with rosacea in the past month. They are mentioned as general dermatologists in this study. The participants in the survey were selected from the registered physicians in m3.com (a medical information website). The sample size was set at 200 based on the number of expected responses. A total of 216 physicians met the eligibility criteria and completed the survey (Figure 2). The online survey of general dermatologists was performed between June 10 and June 24, 2024. The survey included questions regarding respondents' backgrounds and some of the items that reached consensus among the panel members. The items were asked to general dermatologists in the same manner as that in Delphi voting.

## 3 Results

### 3.1 Overview of the Delphi Results

**FIGURE 3:**
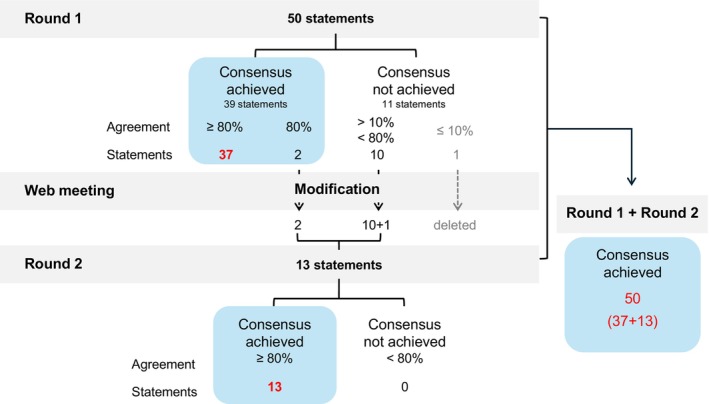
Process of developing a consensus on rosacea‐related contents.

The development process of the consensus items is shown in Figure 3. In the first round, 50 items were targeted, and consensus was obtained for 39 items. The voting results and feedback from the open‐ended questions in the first round were discussed in an online meeting by four panel members involved in designing the Delphi method. Consequently, 10 out of 11 items that did not reach consensus in Round 1 were revised. Two items that reached consensus were also revised based on members' opinions that were expressed in the open‐ended section. In relation to these, the following item was newly added: “When diagnosing rosacea, atopic dermatitis and hay fever should be excluded in patients whose chief complaint is a red face.” The only remaining item (“Oral administration of glucocorticoids is effective in the treatment of rosacea”) failed to reach consensus, resulting in only 10% agreement in Round 1. According to the panel members, although a transient effect can be expected because of the anti‐inflammatory action of glucocorticoids, there is a possibility of exacerbation after their discontinuation and that there is a lack of experience and evidence regarding the oral administration of glucocorticoids for rosacea. Thus, we considered that the oral administration of glucocorticoids should be discussed when adequate evidence is developed and deleted this item. Hence, Round 2 included 13 items, and consensus was reached for all items. Altogether, a total of 50 items reached consensus. The detailed results of each Delphi round are shown in Tables S1 and S2.

### 3.2 Consensus Statements

The consensus statements obtained via the Delphi method are shown in Sections I–IV. The Delphi voting results for each item are shown in parentheses (e.g., “9/10” indicates that 9 out of 10 members voted “agree”). The following sections include the contents that panel members considered important for each item. The original text of the consensus items is shown in Table S3.

#### 3.2.1 Section I. Types of Rosacea and Their Characteristics

The consensus statements for Section I are shown in Table 1. The panel members discussed the skin condition of patients with rosacea at the postsurvey meeting. Moreover, 80% of the members agreed with the following item: “The skin of patients with erythematotelangiectatic rosacea may be dry, which is often accompanied with decreased moisture content of the stratum corneum.” However, some expressed that oily skin was more common than dry skin.

**TABLE 1:** Consensus statements Section I. Types of rosacea and their characteristics.

Consensus statements
There are four basic types of rosacea: erythematotelangiectatic, papulopustular, phymatous/rhinophyma, and ocular, but they are often intermixed (*n* = 10/10).
Erythematotelangiectatic rosacea is a type of rosacea wherein the primary symptoms include erythema and telangiectasia caused by inflammation around sebaceous follicles (*n* = 10/10). The skin of its patients may be dry, which is often accompanied with decreased moisture content of the stratum corneum (*n* = 8/10). Their skin is also accompanied with itching, discomfort, burning, and stinging sensation (*n* = 9/10).
Papulopustular rosacea is a type of rosacea wherein the primary symptoms include papule and pustule formation centered on sebaceous follicles (*n* = 10/10). Although the presence or absence of vasodilation is not essential for diagnosing papulopustular rosacea, it is associated with a high rate of vasodilation (*n* = 9/10). The skin of its patients is accompanied with itching, discomfort, burning, and stinging sensation (*n* = 9/10).
Phymatous rosacea/rhinophyma is a type of rosacea wherein the primary symptom is mass formation because of inflammation and fibrosis of the dermis (*n* = 10/10).
Ocular rosacea is a type of rosacea wherein the primary symptom is inflammation extending from the eyelid to the eyelid conjunctiva and ocular conjunctiva because of inflammation around the meibomian glands (*n* = 9/10).
2 The characteristics of rosacea include flushing, transient/persistent erythema, telangiectasia, papules, and pustules. These are accompanied by sensory symptoms, including itching, discomfort, burning, stinging sensation, and edema.
A transient increase in redness, described as flushing and transient erythema in rosacea, can be induced by various triggers (*n* = 10/10), and they can be accompanied by heat sensation, hot flash, burning, or pain (*n* = 9/10). Those symptoms in rosacea occur on the glabella, chin, cheeks, etc. (*n* = 9/10), but not the trunk in principle (*n* = 8/10).
Persistent erythema in rosacea worsens in response to various triggers (*n* = 10/10).
Telangiectasia of rosacea is a visible dilation of blood vessels that occur on the glabella, chin, cheeks, etc. (*n* = 9/10). It should be differentiated from capillary vasodilation caused by the use of topical glucocorticoids, ultraviolet damage, or aging (*n* = 8/10).
Rosacea papules are red papules (which can reach a size up to a half grain of rice) that may be accompanied by pustules and that are usually seen on the glabella, chin, cheeks, nose, etc. (*n* = 10/10). The papules and pustules of rosacea are clinically similar to those of acne, but are not accompanied by comedones unless acne is present (*n* = 9/10).
The burning or stinging sensation associated with rosacea occurs primarily in the cheeks, but may also occur in the mandible or forehead area (*n* = 10/10).
The skin of patients with rosacea may be accompanied with acute or chronic localized edema (*n* = 9/10). Acute edema associated with rosacea may last for several days (*n* = 10/10).

*Note:* The data in parentheses indicate the Delphi voting results.

The following statement was discussed: “Telangiectasia of rosacea should be differentiated from capillary vasodilation caused by the use of topical glucocorticoids, ultraviolet damage, or aging.” Because several factors are involved in telangiectasia on the face of individuals with rosacea, opinions regarding the possibility of complete differentiation and the necessity of differentiation differed among the expert panelists.

#### 3.2.2 Section II. Differential Diagnosis and Treatment of Rosacea

The consensus statements for Section II are shown in Table 2. Eighty percent or more of the panel members agreed with the following statements: “Perioral dermatitis is an independent disease distinct from rosacea” and “Steroid‐induced rosacea is synonymous with rosacea‐like dermatitis in Japan.” However, they emphasized the need for clearer definitions and classifications, considering the lack of distinction between rosacea exacerbated by glucocorticoids and dermatitis induced by glucocorticoids.

**TABLE 2:** Consensus statement Section II. Differential diagnosis and treatment of rosacea.

Consensus statements
In rosacea treatment, it is important to determine the type of disease and to select treatment methods according to the syndrome and type of disease (*n* = 10/10). If there are skin features suggestive of rosacea, it is helpful in the differential diagnosis to check for associated symptoms outside of the face (*n* = 10/10). In addition, underlying diseases and comorbidities should also be considered (*n* = 10/10).
2 Rosacea may also occur in combination with atopic dermatitis or contact dermatitis (pollen dermatitis) (*n* = 9/10). Thus, when diagnosing rosacea, atopic dermatitis and hay fever should be excluded in patients whose chief complaint is a red face (*n* = 10/10). If there is a complication of atopic dermatitis (*n* = 10/10) or hay fever (*n* = 10/10), treatment priority should be considered according to the degree of symptoms (and exacerbating factors in the case of hay fever).
3 In papulopustular rosacea, *Demodex folliculorum* may be identified but treated as rosacea (*n* = 9/10).
4 In differentiating rosacea from other diseases, the presence or absence of erythema around pores and telangiectasia is a key factor in judgment (*n* = 9/10). Steroid‐induced rosacea is caused by the inappropriate use of topical glucocorticoids and other drugs, and is synonymous with rosacea‐like dermatitis in Japan (*n* = 9/10). On the other hand, perioral dermatitis is a disease that causes red papules and/or erythema with pustules around the mouth and nasolabial folds, and is an independent disease distinct from rosacea (*n* = 8/10).

*Note:* The data in parentheses indicate the Delphi voting results.

Moreover, the panelists discussed the involvement of *Demodex* mite infestation in the pathogenesis of rosacea. *Demodex* is often identified in rosacea. The initial inflammation of rosacea promotes *Demodex* infestation, which in turn exacerbates the disease. In addition to this secondary event, *Demodex* may contribute to the early inflammatory process. The panel members also stated that *Demodex* is one of the factors that exacerbate rosacea symptoms and that after the conditions for rosacea are in place, the environment may become more conducive to increasing the number of mites.

#### 3.2.3 Section III. Factors That Trigger or Worsen Rosacea Symptoms and Disease Management

The consensus statements for Section III are shown in Table 3. In the present study, the panel members did not reach consensus regarding the statement by ROSCO, “In the treatment of rosacea, it is important to aim for clear, symptom‐free skin (Investigator's Global Assessment; IGA 0).” Compared with Europe and the United States, aiming for IGA 0 may not be currently possible in Japan because few treatment options covered by insurance are available in Japan. Instead, it is desirable to maintain a condition wherein the patient's daily life is not affected by symptoms.

**TABLE 3:** Consensus statement Section III. Factors that trigger or worsen rosacea symptoms and disease management.

Consensus statements
The onset and recurrence of rosacea may involve environmental factors, and genetic predisposition may also play a role (*n* = 8/10). Especially, exacerbating factors for Japanese patients with rosacea include not only the typical factors (e.g., sun exposure, temperature differences, and intense stress), but also pollen and menstrual cycles (*n* = 9/10). Besides, the use of topical glucocorticoids in red‐faced patients with a predisposition to rosacea can exacerbate the disease as steroid rosacea (*n* = 9/10).
2 Rosacea is an intractable disease; therefore, the goal of treatment should be the long‐term maintenance of a condition wherein the patient's daily life is not affected by symptoms rather than complete cure (*n* = 10/10). To accomplish the goal, it is necessary to follow the evolution of symptoms and changes in their impact on daily life with the patient as treatment progresses (*n* = 10/10). Therefore, it is important to explain to patients and have them understand that although a “cure” (a condition that does not require treatment) cannot be achieved, symptoms can be controlled through treatment and appropriate skin care (*n* = 10/10). Additionally, when examining a patient with rosacea, consideration must be given not only to external factors (makeup, ultraviolet rays, dryness, scratching, infection, etc.) and physical aspects (e.g., skin symptoms), but also to psychological and social aspects (living environment aspect) (*n* = 10/10). In this regard, the DLQI (Dermatology Life Quality Index) is an index that can assess the quality of life of patients with rosacea (*n* = 8/10).
3 There is a lack of simple and appropriate indicators to assess the severity of rosacea (*n* = 9/10), as well as treatment satisfaction of patients with rosacea (*n* = 9/10).

*Note:* The data in parentheses indicate the Delphi voting results.

#### 3.2.4 Section IV. Treatment and Patient Guidance for Rosacea

The consensus statements for Section IV are shown in Table 4. The panel members discussed the effectiveness of the Sulfur and Camphor Lotion. Since the 1970s, the Sulfur and Camphor Lotion, which exerts keratolytic and degreasing effects, has been used to treat rosacea under national health insurance in Japan [8]. Recently, topical sulfur or metronidazole preparation have been reported to have comparably favorable effects for both erythematotelangiectatic rosacea and papulopustular rosacea in Japanese patients [17]. However, clinical studies evaluating the efficacy of the Sulfur and Camphor Lotion as a treatment for rosacea are considerably limited. Owing to this background, the panel members concluded that further evidence is essential to support the active use of the Sulfur and Camphor Lotion in general clinical practice.

**TABLE 4:** Consensus statement Section IV. Treatment and patient guidance for rosacea.

Consensus statements
Guidance for patients with rosacea
Patients with rosacea should be instructed to cleanse with hypoallergenic cleansers and moisturize appropriately for their skin type (*n* = 9/10).
Patients with rosacea whose exacerbating factor is sun exposure should be instructed to use hypoallergenic sunscreens and to shield themselves from the sun with hats, parasols, etc. to prevent their disease from worsening because of sun exposure (*n* = 9/10).
2 Treatment of erythematotelangiectatic rosacea
Physical therapy with IPL (intense pulsed light) or dye laser should be considered for erythematotelangiectatic rosacea that does not improve with skin care instructions or removal of exacerbating factors (*n* = 10/10).
Topical metronidazole is unlikely to have a therapeutic effect on telangiectasias in erythematotelangiectatic rosacea but may have a therapeutic effect on erythema associated with the inflammation of sebaceous follicles (*n* = 10/10).
3 Treatment of papulopustular rosacea
Treatment of papulopustular rosacea is effective with oral administration of tetracyclines (doxycycline, minocycline, and tetracycline) (*n* = 9/10), as well as topical metronidazole (*n* = 10/10).
The Sulfur and Camphor Lotion is covered by insurance in Japan for the treatment of papulopustular rosacea and may be effective, but can cause exacerbation by stimulation (*n* = 9/10).
4 Treatment of ocular rosacea
In ocular rosacea, consultation with an ophthalmologist is necessary at least once because of the possibility of severe disease (*n* = 9/10).
5 Treatment of rosacea‐like diseases
Topical metronidazole is an option for steroid rosacea (*n* = 8/10).

*Note:* The data in parentheses indicate the Delphi voting results.

### 3.3 Extra Research: Online Survey of General Dermatologists

An online survey was performed to understand the current awareness of rosacea among general Japanese dermatologists. The general dermatologists (*n* = 216) answered a questionnaire concerning the consensus items that were identified by the expert panel. The physician background is shown in Table 5. The list of 50 items was narrowed down to 22 items, which formed the basis for diagnosing and treating rosacea. The participants indicated the degree of agreement for these items. As shown in Table 6, ≥ 80% of the dermatologists agreed on 13 of the 22 items. The agreement percentages for the remaining nine items ranged from 51.4% to 78.2%. Four out of the nine items with low agreement percentages are from “Differential diagnosis and treatment of rosacea” (agreement percentage: 59.7%–70.8%) in Section II and “Treatment and patient guidance for rosacea” (agreement percentage: 63.0%–78.2%) in Section IV. Moreover, three out of the nine items with low agreement percentages were related to perioral dermatitis and steroid‐induced rosacea or steroid rosacea. The following item had the lowest level of agreement: “The skin of patients with erythematotelangiectatic rosacea may be dry, which is often accompanied with decreased moisture content of the stratum corneum” (51.4%).

**TABLE 5:** Demographics of general dermatologists who participated in the online survey.

	*n* = 216
Age (years), *n* (%)	
20–29	3 (1.4)
30–39	44 (20.4)
40–49	62 (28.7)
50–59	61 (28.2)
60 and above	46 (21.3)
Board‐certified dermatologist by the Japanese Dermatological Association, *n* (%)	
Yes	157 (72.7)
No	59 (27.3)
Main department, *n* (%)	
Dermatology	199 (92.1)
General internal medicine/general medicine	0 (0.0)
Allergy	0 (0.0)
Pediatrics	0 (0.0)
Plastic surgery	17 (7.9)
Others	0 (0.0)
Main affiliated facilities, *n* (%)	
University hospital	43 (19.9)
National/public hospital	26 (12.0)
Other general hospitals	45 (20.8)
Clinic/medical office	102 (47.2)
Others	0 (0.0)
Number of patients with rosacea treated in the last month	
Median (1st quartile, 3rd quartile)	7.5 (5,15)

**TABLE 6:** Statements presented in the online survey of general dermatologists and their responses.

	Items	Disagree (*n*)	Neither agree nor disagree (*n*)	Agree (*n*)	Don't know/can't answer (*n*)	Agreement (%)
1	There are four basic types of rosacea: erythematotelangiectatic, papulopustular, phymatous/rhinophyma, and ocular, but they are often intermixed.	2	9	203	2	94.0
2	A transient increase in redness, described as flushing and transient erythema in rosacea, can be induced by various triggers.	1	7	204	4	94.4
3	The skin of patients with erythematotelangiectatic rosacea may be dry, which is often accompanied with decreased moisture content of the stratum corneum.	13	71	111	21	51.4
4	Rosacea may also occur in combination with atopic dermatitis or contact dermatitis (pollen dermatitis).	8	48	153	7	70.8
5	The use of topical glucocorticoids in red‐faced patients with a predisposition to rosacea can exacerbate the disease as steroid rosacea.	1	16	199	0	92.1
6	Exacerbating factors for Japanese patients with rosacea include not only the typical factors (e.g., sun exposure, temperature differences, and intense stress), but also pollen and menstrual cycles.	4	24	184	4	85.2
7	Perioral dermatitis is a disease that causes red papules and/or erythema with pustules around the mouth and nasolabial folds, and is an independent disease distinct from rosacea.	15	63	129	9	59.7
8	In rosacea treatment, it is important to determine the type of disease and to select treatment methods according to the syndrome and type of disease.	1	35	177	3	81.9
9	If there are skin features suggestive of rosacea, underlying diseases and comorbidities should also be considered.	3	25	186	2	86.1
10	Steroid‐induced rosacea is caused by the inappropriate use of topical glucocorticoids and other drugs, and is synonymous with rosacea‐like dermatitis in Japan.	13	44	152	7	70.4
11	Physical therapy with IPL (intense pulsed light) or dye laser should be considered for erythematotelangiectatic rosacea that does not improve with skin care instructions or removal of exacerbating factors.	5	58	136	17	63.0
12	In rosacea patients with a red face as the main complaint, if there is a complication of atopic dermatitis, treatment priority should be considered according to symptom severity.	0	21	191	4	88.4
13	In rosacea patients with a red face as the main complaint, if there is a complication of hay fever, treatment priority should be considered according to the degree of symptoms and exacerbating factors.	0	29	181	6	83.8
14	There is a lack of simple and appropriate indicators to assess the severity of rosacea.	5	35	173	3	80.1
15	Rosacea is a chronic disease. Therefore, it is necessary to follow the evolution of symptoms and changes in their impact on daily life with the patient as treatment progresses.	0	12	204	0	94.4
16	In papulopustular rosacea, *Demodex folliculorum* may be identified but treated as rosacea.	21	44	141	10	65.3
17	Patients with rosacea whose exacerbating factor is sun exposure should be instructed to use hypoallergenic sunscreens and to shield themselves from the sun with hats, parasols, etc. to prevent their disease from worsening because of sun exposure.	1	16	197	2	91.2
18	Treatment of papulopustular rosacea is effective with oral administration of tetracyclines (doxycycline, minocycline, and tetracycline).	1	27	183	5	84.7
19	Topical metronidazole is unlikely to have a therapeutic effect on telangiectasias in erythematotelangiectatic rosacea but may have a therapeutic effect on erythema associated with the inflammation of sebaceous follicles.	4	37	169	6	78.2
20	Topical metronidazole is effective in the treatment of papulopustular rosacea.	1	25	185	5	85.6
21	Topical metronidazole is an option for steroid rosacea.	12	42	154	8	71.3
22	In ocular rosacea, consultation with an ophthalmologist is necessary at least once because of the possibility of severe disease.	4	39	150	23	69.4

## 4 Discussion

In this study, an expert panel in Japan developed a consensus statement related to rosacea (e.g., disease types, characteristics, diagnosis, factors for onset and exacerbation, and treatment) using a similar method to that of ROSCO. Altogether, the panel members reached consensus on 50 items. Moreover, 22 of these items were presented to general dermatologists in an online survey. At least 80% of the general dermatologists agreed on 13 of them. With regard to the 9 items for which consensus was not reached, the agreement percentages for items in Section II “Differential diagnosis and treatment of rosacea” were relatively low (70.8% or less for all items), indicating the need to clearly define and organize the respective diagnostic terms for rosacea and its related conditions. In the postsurvey meeting, final agreement was confirmed among the panel members on each consensus statement. Moreover, the meeting covered six topics that were considered important and required further discussion by the panel members. In this section, we describe the current state of rosacea diagnosis and treatment in Japan, including the content of the postsurvey meeting.

### 4.1 Ocular Rosacea Symptoms

The panel members reached consensus on the item “Ocular rosacea is a type of rosacea wherein the primary symptom is inflammation extending from the eyelid to the eyelid conjunctiva and ocular conjunctiva because of inflammation around the meibomian glands.” They stated that this symptom is often observed when ocular rosacea is combined with papulopustular rosacea. Moreover, based on their clinical experience, an opinion was raised that the case wherein granulomatous changes like those seen in lupus miliaris disseminatus faciei occur on the eyelids is more likely to be observed when it is present in combination with erythematotelangiectatic rosacea. However, few studies have focused on these symptoms. Thus, it is important to clarify the symptoms of ocular rosacea combined with other rosacea. Taking previous reports [7, 18, 19] into consideration as well, the panel members recommend consultation with ophthalmologists to exclude other ocular diseases.

### 4.2 Characteristics of the Skin of Patients With Rosacea

Some panel members indicated that many patients with rosacea experience oily skin as well as dry skin. With regard to water levels, several studies have shown that patients with rosacea exhibit lower stratum corneum water content and higher transepidermal water loss in the facial epidermis compared with healthy individuals [20, 21, 22]. On the other hand, previous research on sebum levels in patients with rosacea has yielded inconsistent results, with some studies reporting no significant difference in sebum levels and composition between patients with rosacea and healthy individuals [23] and others observing lower sebum levels in patients with papulopustular rosacea [24]. In addition, one study found that rosacea patients with lesions limited to the nose had higher nasal oil levels than healthy participants [21]. In clinical practice, some patients may exhibit excessive sebum secretion, resulting in an oily and shiny appearance. In summary, although patients with rosacea typically exhibit lower stratum corneum water content and higher transepidermal water loss, higher sebum levels may also be observed depending on the characteristics of individual patients.

### 4.3 Differentiating Between Rosacea and Similar Diseases

**FIGURE 4:**
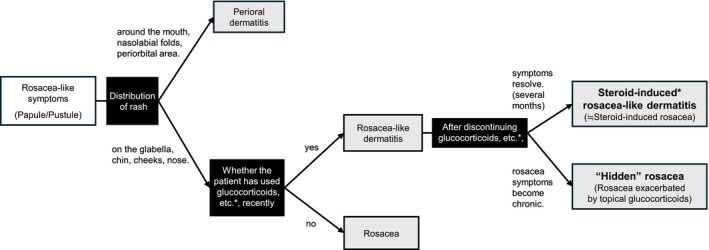
Diagnosis of patients with rosacea‐like symptoms. The diagram shows the proposed diagnostic process when rosacea‐like symptoms (e.g., papules or pustules) are detected. Black squares indicate the key points for diagnosis, whereas gray squares indicate the results of the diagnosis, including some tentative results. *There are reports of rosacea being triggered by drugs, not just glucocorticoids, but also calcineurin inhibitors, etc.

Although consensus was reached with regard to the definitions and differentiation of steroid‐induced rosacea, rosacea‐like dermatitis, perioral dermatitis, and rosacea, the complexity of differentiating these conditions was one of the main discussion points at the postsurvey meeting. This challenge began in the 1960s when the term “perioral dermatitis” was first reported [25]. Perioral dermatitis is a polymorphous eruption that mainly involves the cutaneous portions of the upper lip, chin, and nasolabial folds. It mostly occurs in young women aged 20–40 years and may also occur in men and children [25]. Thus, perioral dermatitis was thought to be different from rosacea. Later, symptoms resembling perioral dermatitis were reported in Germany, which were referred to as “rosacea‐like dermatitis” [26]. Hence, to date, “perioral dermatitis” and “rosacea‐like dermatitis” are often lumped together. In 1969, Sneddon suggested that the treatment using glucocorticoids partially led to an increase in rosacea‐like dermatitis [27]. Various names have since been assigned by different researchers to describe this glucocorticoid‐induced rosacea‐like dermatitis, including steroid‐induced rosacea and “iatrosacea” [28]. Subsequently, in the current prominent dermatology textbooks, steroid rosacea is defined as the worsening of rosacea in patients due to the topical application of glucocorticoids [29], whereas steroid‐induced rosacea is defined as the development of rosacea‐like symptoms resulting from the use of topical glucocorticoid medications, which resolves with treatment [30]. However, the term “steroid rosacea” is not widely used, similar to how generally accepted rosacea triggers, such as UV radiation and temperature fluctuations, are not typically included alongside the disease name. Given these facts, it is thought that there is no consensus regarding the definition and designation of the skin conditions induced by glucocorticoid use. Similarly, in Japan, no strict distinction has been made between steroid rosacea and steroid‐induced rosacea. Furthermore, specifically in Japan, these conditions are widely accepted as equivalent to rosacea‐like dermatitis in cases that rosacea‐like symptoms are evoked after glucocorticoid use. This is believed to partly stem from the initial emphasis on the occurrence of perioral and rosacea‐like dermatitis as side effects of glucocorticoids when these classifications were first introduced in Japan [31]. Moreover, the limited availability of effective drug options for rosacea in clinical practice, until a few years ago, reduced the importance of detailed classification. In the present study, many panel members agreed that rosacea and perioral dermatitis can be differentiated based on the skin rash distribution and that rosacea‐like dermatitis and steroid‐induced rosacea are used as synonymous diagnoses in Japan. However, they had different opinions regarding the details. The most common viewpoint was that patients who develop rosacea‐like symptoms because of the use of glucocorticoids, tacrolimus, or other medications can be categorized into two groups: those who recover after removing the trigger and those who experience recurrent flares even after removing the medications. This difference may be attributed to the individual's predisposition to rosacea. In predisposed individuals, glucocorticoids or tacrolimus may trigger rosacea, complicating its management. However, identifying individuals with a predisposition to rosacea before initiating dermatosis treatments is difficult. Thus, the panel proposed an algorithm for differentiating rosacea in Japan (Figure 4). Rosacea and perioral dermatitis can be differentiated based on the skin rash distribution and age of onset. If it is clear at the initial consultation that glucocorticoids or tacrolimus is not the cause, the patient is diagnosed with rosacea. Conversely, if glucocorticoid or tacrolimus use is confirmed, the condition is tentatively considered rosacea‐like dermatitis, and the responsible agent (such as glucocorticoids or tacrolimus) is discontinued. If the symptoms resolve in a few months following its discontinuation, the patient is classified as steroid‐induced rosacea‐like dermatitis. This term is synonymous with what had previously been recognized as steroid‐induced rosacea. The term “steroid‐induced rosacea‐like dermatitis” is newly proposed to correctly describe the disease, emphasizing that the condition does not originate from a rosacea predisposition; in other words, it is not rosacea. For convenience, the term “steroid‐induced rosacea‐like dermatitis” is used, although in clinical practice, it encompasses symptoms caused by other causative agents such as tacrolimus. Contrarily, if the symptoms do not resolve and recur after the discontinuation of glucocorticoids, etc., the patient is classified as “hidden” or “undiagnosed” rosacea. As described earlier, due to the historical background and limited treatment options available, Japan has not strictly differentiated between steroid‐induced rosacea (dermatitis induced by glucocorticoids, etc.) and steroid rosacea (rosacea exacerbated by glucocorticoids, etc.) within the broader category of rosacea‐like dermatitis. However, with the recent approval of treatment options indicated for rosacea, appropriate management for rosacea has become possible. Consequently, it is now necessary to appropriately differentiate cases of rosacea that may be latent in rosacea‐like dermatitis. The concept of “hidden” rosacea is identical to that of steroid rosacea defined in prominent dermatology textbooks, referring to a state wherein a patient had an underlying rosacea predisposition and the condition was exacerbated by the use of agents such as glucocorticoids. Nonetheless, focusing on the possibility that “true” rosacea patients may be “hidden” among those currently recognized as having rosacea‐like dermatitis, we propose the term “hidden” rosacea, although it is not an established medical term. However, there are a few exceptions to this diagnostic process. For example, perioral dermatitis is occasionally diagnosed as rosacea‐like dermatitis if the rash spreads beyond the mouth area or occurs in older adults. Although we proposed the algorithm for differentiating rosacea in Figure 4, many aspects regarding the pathogenesis, predisposition, and exacerbating factors of rosacea are unclear. In addition, the predisposing factors for rosacea have not been determined. The above algorithm may need to be reviewed and updated as and when the predisposing factors of rosacea are clarified.

### 4.4 Treatment Goal for Rosacea in Japan

The ROSCO panel reached consensus on the following item: “In the treatment of rosacea, it is important to aim for clear, symptom‐free skin (IGA 0)” [7]. However, in the present study, only half of the panel members agreed with this item. Therefore, the statement was revised as follows: “Rosacea is an intractable disease, and the goal of treatment should be the long‐term maintenance of a condition wherein the patient's daily life is not affected by symptoms rather than complete cure.” All panel members agreed with this statement. When planning treatment regimens, drug availability in Japan should be considered. In the United States, the Food and Drug Administration approved azelaic acid, brimonidine tartrate, ivermectin, metronidazole, oxymetazoline hydrochloride, sodium sulfate, sulfur, and calcineurin inhibitors as topical drugs and doxycycline as a systemic drug for rosacea treatment [32]. However, in Japan, only metronidazole and the Sulfur and Camphor Lotion have been approved [8]. Because Japan has fewer treatment options compared with Western countries, dermatologists needed to identify exacerbating factors in individual patients through interviews and share the treatment goal and plan with patients. Tailor‐made patient guidance, including daily life habits and skincare, will facilitate the long‐term maintenance of the disease without affecting the patient's daily life. However, further research and the approval of more drugs are essential to achieving optimal treatment options.

In addition, the Japanese skin type should be considered when planning treatment regimens. Many Japanese people are classified as Fitzpatrick skin phototypes III–V [33] and have relatively darker skin than Caucasians. According to a previous study, identifying erythema and telangiectasia in dark skin is difficult, and there is a possibility of underdiagnosis and delayed diagnosis [12]. The same situation may also be true for the treatment effects. It is possible that the delay in diagnosis and subsequent start of treatment may make it challenging to achieve remission or that the treatment effects are difficult to confirm because they are masked by the patient's skin tone.

Moreover, some panel members commented that the consideration about aiming for IGA 0 largely depends on whether patients prioritize a complete cure. Because treatment has not been established to completely cure rosacea, if patients expect cure of rosacea and if the condition has not been completely cured, the expectation itself could be a source of stress or burden for patients. Moreover, completely removing the exacerbating factors of rosacea for every patient is difficult, depending on the patient's occupation or residential area. Therefore, the panel members aimed to stabilize the skin condition so that rosacea symptoms do not severely affect the patient's quality of life.

In summary, the circumstances within Japan can be considered different from those in Europe and the United States owing to the few treatment options that are available and the difficulty in confirming changes in symptoms because of the darker skin of Japanese patients. Moreover, when considering the demand for individualized treatment goals that account for the patient's environment in actual clinical practice throughout Japan, experts recommended that the goal of rosacea treatment should be to “maintain a state wherein the patient's daily life is not affected by symptoms over the long term” rather than “complete cure,” which is different from the approach adopted in Europe and the United States.

### 4.5 Recent Trends in Rosacea Incidence in Japan

Although the exact prevalence of rosacea in Japan remains unknown, the panel members realized that the number of patients with rosacea has been increasing recently. They associated this increase to two causes: an increase in diagnosis opportunities and an actual increase in the number of patients in Japan. With regard to the former, the panel members interpreted it as follows: dermatologists have become more aware of rosacea since the approval of metronidazole as a drug for treating rosacea; thus, there are more rosacea cases being diagnosed than before. Before its approval, treatment options for rosacea were limited; therefore, medications approved for acne and other dermatoses, which were believed to be potentially effective against rosacea, were sometimes prescribed as symptomatic therapy. The introduction of a new treatment option enhanced the clinical importance of diagnosing rosacea, which may have contributed to the observed increase in the number of patients diagnosed with the condition. With regard to the latter, there was a panelist opinion that dermatologists' drug selection has changed from glucocorticoids to calcineurin inhibitors (e.g., tacrolimus) to avoid adverse reactions to glucocorticoids, resulting in the unintentional exacerbation of rosacea. In Japan today, glucocorticoids are well known to trigger rosacea symptoms. Therefore, tacrolimus is often used in place of glucocorticoids to avoid the side effects of topical glucocorticoids and triggering rosacea. However, the fact that calcineurin inhibitors (e.g., tacrolimus) may also trigger rosacea is not so well known [34]. With the growing interest in rosacea among Japanese dermatologists, the proper use of drugs, including glucocorticoid and calcineurin inhibitors, under adequate diagnosis should be widely publicized.

### 4.6 Current Issues in Rosacea Treatment in Japan

The results of the online survey of general dermatologists may reflect the current situation of rosacea treatment in Japan. Among the 22 items, the largest difference between general dermatologists and panel members in the agreement percentages was observed for three items. The agreement percentage among general dermatologists for the following item was only 63.0% (panel members 100%): “Physical therapy with IPL (intense pulsed light) or dye laser should be considered for erythematotelangiectatic rosacea that does not improve with skin care instructions or removal of exacerbating factors.” In the Japanese guidelines, a pulsed dye laser and IPL are recommended as treatment options for erythematotelangiectatic rosacea [8]. Although various studies have reported the efficacy of laser therapy for rosacea, there are some issues in Japan, including the lack of a uniform treatment protocol, few reports that follow the long‐term prognosis, and difficulty of comparing individual studies because of the different settings for each type of laser therapy devices [8]. These issues may be one of the reasons why general dermatologists have a lower agreement level on this item.

The agreement percentage among general dermatologists for the following item was only 51.4% (panel members 80%): “The skin of patients with erythematotelangiectatic rosacea may be dry, which is often accompanied with decreased moisture content of the stratum corneum.” As previously mentioned, although patients with rosacea mainly have dry skin, there are some patients with high sebum production. The existence of these patients may explain why the above statement focusing on dryness has not been accepted by general dermatologists. In relation to this, the panel members also pointed out that the definition of skin characteristics is vague. Moreover, there are no standard levels of the stratum corneum water content and sebum content. In clinical practice, for dry skin in rosacea, confusion may have arisen from the mixed opinions of general dermatologists, some of whom differentiate between cases with both low water content and low sebum content and cases with low water content and high sebum content, while others do not. The definitions of these terms need to be clarified.

The agreement percentage among general dermatologists for the following item was 65.3% (panel members 90%): “In papulopustular rosacea, *Demodex folliculorum* may be identified but treated as rosacea.” Considering the statement “Although oral ivermectin and metronidazole are considered for the treatment of *Demodex* detected in papulopustular rosacea, there is no high‐quality evidence that can be recommended” in the guideline [8], it is thought that Japanese dermatologists follow the treatment guidelines for rosacea.

### 4.7 Limitations of This Study

This study has several limitations. First, the Delphi method was used to reach a final consensus. Considering the limited clinical studies on rosacea and limited treatment options available in Japanese health care insurance, integrating expert opinions is an appropriate approach to develop consensus statements on rosacea. However, this method does not provide a definitive answer. Second, ophthalmologists were not included in the panel of this study because there are few ophthalmologists who specialize in rosacea and meibomian gland dysfunction in Japan. Ophthalmological expertise is needed to discuss ocular rosacea more precisely. Third, the fundamental data and information from the survey of general dermatologists were limited to descriptive analysis as the main purpose of this study was to establish a consensus on the pathogenesis, diagnosis, treatment, and patient guidance for rosacea. Although this analysis allowed us to understand the perceptions of Japanese dermatologists on each item, further research is needed, including analysis of causal relationships and underlying factors. Finally, this study is the first to establish a consensus on rosacea management in Japan. Therefore, it primarily focuses on the basic content of rosacea and its treatment. The fact that certain aspects are not discussed in this paper does not suggest that they are unimportant.

## 5 Conclusion

In this study, the current knowledge of rosacea was organized by integrating expert opinions on its disease concept, definition, differential diagnosis, treatment, and patient guidance, thereby developing consensus statements for Japan. Recently, rosacea has attracted increasing interest, and it is becoming more widely known, not only among medical professionals. Based on the increased interest in rosacea among academic researchers, updates on the state of the disease and its treatment mechanisms are expected. Moreover, awareness of the present consensus needs to be promoted among dermatologists, including nonspecialists in rosacea, and should be updated as clinical experience and evidence concerning rosacea accumulate. We hope that the consensus established in this study can be utilized to enhance the systematic treatment for rosacea and provide the basis for guidance on treating rosacea in Japan.

## Funding

The planning and delivery of this project were funded by Maruho Co., Ltd., which engages in the manufacturing and sale of metronidazole gel (Rozex Gel 0.75%). The sponsor was not involved in the voting, discussion, or handling of data.

## Ethics Statement

The authors have nothing to report.

## Conflicts of Interest

K.Y. is a member of the Editorial Board of the Journal of Dermatology. To minimize bias, K.Y. was excluded from all editorial decision‐making related to the acceptance of this article for publication. K.Y. has received consulting fees from POLA Chemical Industries Inc., Sato Pharmaceutical Co., Ltd., Nippon Boehringer Ingelheim Co., Ltd., ROHTO Pharmaceutical Co., Ltd.; and honoraria for lectures from Maruho Co., Ltd., Sato Pharmaceutical Co., Ltd., The Estée Lauder Companies Inc., LEO Pharma K.K., Grafa Laboratories Co., Ltd., Kracie Ltd., ROHTO Pharmaceutical Co., Ltd., but none of them affect the contents of the article. N.H. has received honoraria for lectures from Maruho Co., Ltd. H.H. has received honoraria for lectures, research grants, and fellowships from Maruho Co., Ltd. Y.F. has no conflicts to declare. Y.H. has received research grants from Maruho Co., Ltd. M.K. has no conflicts to declare. Y.N. has received research grants from Maruho Co., Ltd., TAIHO Pharmaceutical Co., Ltd., and Alexion Pharmaceuticals Inc.; and fellowships from Maruho Co., Ltd., TAIHO Pharmaceutical Co., Ltd., and Alexion Pharmaceuticals Inc. J.O. has received research grants and fellowships from Maruho Co., Ltd. K.T. has received honoraria for lectures, research grants, and consulting fees from Maruho Co., Ltd., and UCB Co., Ltd. These financial relationships had no influence on the content of this article. Y.M. has received honoraria for lectures, manuscript fees, research grants, and fellowships from Maruho Co., Ltd.

## Supporting information




**Table S1:** Voting results of the Delphi method (Round 1).
**Table S2:** Voting results of the Delphi method (Round 2).
**Table S3:** Fifty items that reached consensus from the panel members.

## Data Availability

Research data are not shared; however, supplementary material is available in the online Supporting Information section of this article.
